# A novel polysaccharide/zein conjugate as an alternative green plastic

**DOI:** 10.1038/s41598-023-40293-4

**Published:** 2023-08-12

**Authors:** Marwa Tallawi, Danial Amrein, Gerd Gemmecker, Katerina E. Aifantis, Klaus Drechsler

**Affiliations:** 1 Carbon Composite, School of Engineering and Design, Technical University of Munich, 85748 Garching, Germany; 2 School of Natural Sciences, Bavarian NMR Center, Technical University of Munich, 85748 Garching, Germany; 3 Department of Mechanical and Aerospace Engineering, University of Florida, Gainesville, FL 32611 USA

**Keywords:** Materials science, Soft materials, Polymers

## Abstract

The flax seed cake is a waste product from flax oil extraction. Adding value to this wasted material aligns with the concept of circularity. In this study, we explored zein protein conjugation with flax mucilage for packaging material development. Although both flax mucilage and zein have excellent film-forming properties, they lack the required mechanical properties for industrial processing and are sensitive to high humidity. We present a simple and non-toxic one-pot method for developing the novel flax mucilage/zein conjugate. Where the flax mucilage undergoes oxidation to form aldehyde groups, which then react with zein's amino groups in a glycation process. The conjugates were analyzed using different techniques. The flax mucilage conjugate had a water-holding capacity of 87–62%. Increasing the zein content improved the surface smoothness of the films. On the other hand, higher levels of zein led to a significant decrease in film solubility (p < 0.05). The flax mucilage conjugate exhibited thermoplastic and elastic properties; revealing Young's modulus of 1–3 GPa, glass transition temperature between 49 °C and 103 °C and excellent processability with various industrial techniques. Showing its potential as a sustainable alternative to traditional plastics.

## Introduction

The field of polymer science has seen remarkable progress since the discovery and advances in petrochemical sciences. Plastics, with their unique and advantageous properties, including high strength-to-weight ratio, stiffness, toughness, ductility, corrosion resistance, and relatively low cost, have become ubiquitous. However, the production and transportation of these materials have contributed significantly to CO_2_ emissions, resulting in a considerably high carbon footprint^1,2^. Moreover, plastics are either incinerated at their end of life releasing more CO_2_ or accumulating in landfills and oceans, posing a significant environmental threat^3,4^. As a result, the disposal of plastics has become a major concern for polymer scientists^5–8^.

To shift towards a Circular Economy and reduce our reliance on fossil fuels, it is crucial to substitute oil-based plastics with sustainable bioplastics derived from polysaccharides, proteins, or lipids of various plants and organisms. Examples of such biopolymers include starch^9,10^ and cellulose^11,12^, soya protein^13–15^, pectin^16,17^, casein^18^, whey protein (WP)^19^, alginate^5,20,21^, gelatin^22^, chitin and chitosan^23,24^, and keratin^25^. Of particular interest are plant-based bioplastics, which offer a two-fold advantage by absorbing CO_2_ during their growth through photosynthesis and integrating and degrading easily into the environment at the end of their life cycle, resulting in a reduced carbon footprint compared to their petroleum-based counterparts^2^.

One of the main concerns associated with plant-based bioplastics is competition with primary human food resources, leading to an increase in food prices and deforestation, similar to plant-based bio-gas^26^. To avoid such issues and achieve a bio-economy, bioplastics should be derived from plant and animal residues, allowing for better waste management and providing additional value.

Polysaccharides are versatile molecules that contain different functional groups, including hydroxyl, amino, carboxylic acid, and aldehydes, making them ideal for conjugation. Polysaccharide–protein conjugates have emerged as a potential sustainable bioplastic material due to their enhanced solubility, emulsion, hydrophobicity, barrier, and mechanical properties, surpassing those of individual polysaccharides and proteins. These conjugates have been extensively researched, developed, and applied in biomedical fields as drug carriers, wound dressings, and emulsifiers in food.

Despite their effectiveness and advances in various other fields, limited research has been conducted on polysaccharide–protein conjugates and their application as an alternative bioplastic for packaging, indicating a need for further research in this area^27,28^. One study aimed to use conjugates of different polysaccharides with whey proteins (WP) for packaging applications via physical (intermolecular) interactions. The resulting conjugates showed higher tensile strength and lower oxygen permeability compared to both neat polysaccharides and WP films^29^. Electrostatic complexes or cross-links were believed to be formed between the polysaccharide and protein structure^30,31^. Investigation of different blends of WP-hydroxypropylmethylcellulose (HPMC) also showed improved mechanical and oxygen barrier properties^32,33^. In another study, WP-Okra polysaccharide (OP) blends demonstrated higher oxygen barrier, water vapor permeability, and enhanced flexibility compared to neat WP or OP films alone, confirming a unique interaction between the WP and OP^34^. Furthermore, the conjugation of three proteins (WP isolate, bovine gelatin, and chicken gelatin) on whole potato flour was investigated to enhance the mechanical properties of potato flour films^35^. Aminated polysaccharides were also synthesized via the covalent bonding of putrescine (1,4-diaminobutane) to alginate and low-methoxyl pectin, revealing the effectiveness of the chemical bonding of pectin–putrescine and alginate–putrescine^27^. The resulting polysaccharide–putrescine conjugates exhibited significantly improved mechanical and chemical properties compared to unmodified, non-covalently bonded polysaccharides and putrescine. These studies open a new chapter for biodegradable/edible flexible films for food packaging applications with enhanced mechanical properties and moisture barrier^27^.

In this study, we present a novel approach to developing a sustainable and environmentally friendly alternative plastic material by synthesizing a polysaccharide–protein conjugate based on flax-mucilage/protein. Mucilage is a hydrocolloid extracted from various parts of plants, such as seeds, leaves, and roots^36^, that can provide blends of different polysaccharides^37^. With its hydrophilic nature, mucilage forms a gel-like aqueous solution with high water absorption capacity and enhanced barrier properties^38^. Flax mucilage, in particular, is known for its excellent film-forming properties, making it a promising candidate for this study. Flaxseed mucilage is composed primarily of arabinoxylan and rhamnogalacturonan I, found in the seed coat's epidermal cell layer, which becomes viscous upon contact with water^39^. To the authors’ knowledge, flax-mucilage/protein conjugates have not been studied in depth and especially as a plastic alternative material.

## Results

### Flax mucilage extraction

**Figure 1:**
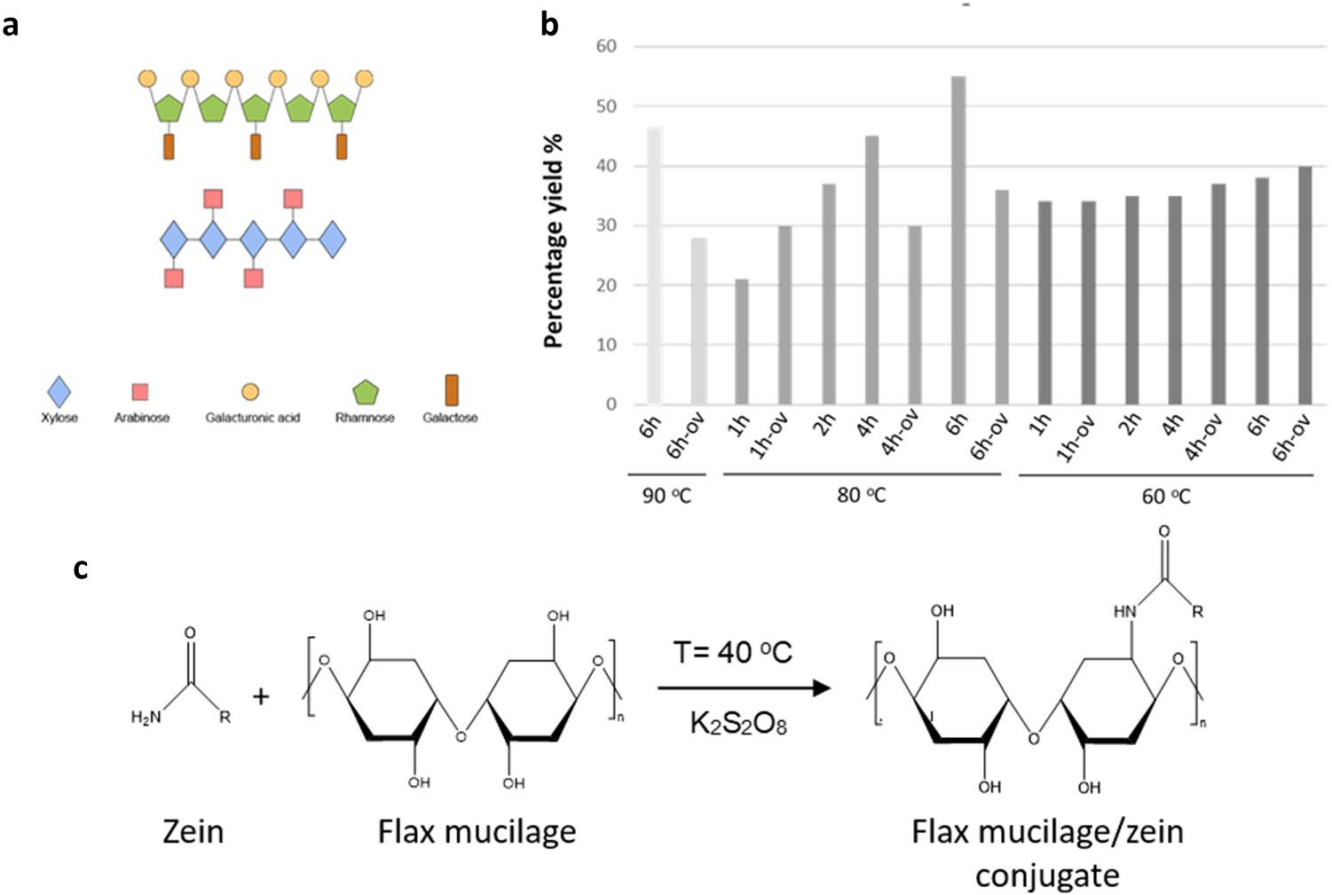
(**a**) Schematic for the flax mucilage composition. (**b**) Flax mucilage extraction yield at different extraction conditions. (**c**) Chemical schematic of the proposed reaction for the conjugation of zein and flax mucilage.

Flax mucilage is a hydrocolloid that can dissolve in water and is mainly composed of arabinoxylan and rhamnogalacturonan I (as shown in Fig. 1a). In our study, we extracted the flax mucilage using the hot water method and then precipitated it with ethanol. We found that the extracted mucilage accounted for 21–55% of the total weight of the initial flaxseed cake residue used, as determined through gravimetric analysis. Interestingly, we observed that increasing the extraction temperature and incubation time led to higher flax mucilage yields. The highest yield of 55% was obtained at 80 °C for 6 h, while the lowest yield of 21% was obtained at 80 °C for 1 h (see Fig. 1b). However, it's important to note that longer periods of heating at high temperatures (~ 80°C to 90°C) may cause thermal degradation of the extracted mucilage structure, as observed by the decrease in yield for the overnight incubation, as shown in Fig. 1b.

The extraction of the flax mucilage has two-fold advantages. Firstly, it does not compete with food resources, and secondly, the utilization of waste byproducts aligns with the concept of circular economy and adds value to agricultural waste. The extracted yield in our study was higher than that obtained from whole flax seeds demonstrated in other studies to yield 5.3–6.2%^40^. This can be attributed to the fact that we used a waste byproduct of the oil industry (flaxseed cake residue) after the oil extraction process, where the oil makes up about 35–45% of the total weight^41^.

### Conjugation of flax mucilage/zein

We utilized potassium persulfate (K_2_S_2_O_8_) as an oxidation method to conjugate the extracted flax mucilage with zein protein. The speculated reaction involving the oxidation and conjugation of zein and flax mucilage are illustrated in Fig. 1c. Various samples were prepared by varying the zein content (5, 10, 20, and 30 w/v %) and reaction times (3 h, 48 h, and 5 days), denoted as 5%-3 h, 5%-48 h, 5%-5D, 10%-3 h, 20%-3 h, and 30%-3 h, respectively. In addition, 30%-3 h was post-treated with Dimethyl Sulfoxide (DMSO) which was referred to as 30%-3 h-DMSO. The average yield of all experiments was approximately 80%, likely due to the loss of some samples during the rinsing step. The yield was calculated based on the initial dry mass of the extracted mucilage and zein used, and the difference between the total dry mass after the reaction and the initial starting material. Our findings show that increasing the zein content resulted in a notable increase in overall yield, with the highest yield observed for sample 30%-3 h (84.72 ± 2.95). Additionally, longer reaction times led to higher yields, showing 75.37 ± 2.72% yield for sample 5%-5D in comparison to 5%-3 h showing only 63.30 ± 2.71%. Additionally, it should be noted that the samples had a high water-holding capacity (as shown in Table 1), with sample 5%-5D exhibiting the highest water-holding capability (approximately 87.61 ± 0.51%) compared to sample 30%-3 h (approximately 62.73 ± 0.07%), which exhibited the lowest. The water-holding capacity of the samples decreased before drying with decreasing reaction time and increasing zein content, indicating fewer functional moieties and surface exposure for water absorption. This may be due to the unreacted functional moieties of zein. The detailed results for yield and water-holding capacity are presented in Table 1.

**Table 1:** Biopolymer properties and surface characterizations from different synthesis conditions.

Sample	Dry yield (%)	Water-holding capacity (%)	Contact angle (°)	Contact angle changes over time (°)	Free surface energy		
					Polar (mN/m)	Dispersive (mN/m)	Total (mN/m)
Zein	–	–	58.97 ± 10.84	–	17.45 ± 6.59	30.52 ± 3.56	47.97 ± 6.09
Flax mucilage	–	–	72.22 ± 3.21	29.37 (5 min) Dissolved (10 min)	–	–	95.3 ± 1.80^43^
5%-5D	75.36 ± 2.72	87.61 ± 0.51	58.45 ± 8.49	–	16.82 ± 4.88	31.89 ± 4.24	48.70 ± 6.02
5%-2D	61.77 ± 7.83	86.54 ± 0.22	54.82 ± 5.40	–	18.84 ± 4.02	32.27 ± 4.18	51.11 ± 3.83
5%-3 h	63.30 ± 2.71	79.36 ± 6.14	55.46 ± 3.52	–	15.51 ± 4.39	31.19 ± 1.68	46.70 ± 4.25
10%-3 h	65.07 ± 7.87	66.24 ± 1.27	58.27 ± 7.42	–	15.45 ± 4.91	35.21 ± 3.56	50.66 ± 4.39
20%-3 h	76.00 ± 1.93	65.21 ± 0.15	57.89 ± 11.25	–	25.24 ± 9.91	28.50 ± 7.14	53.74 ± 8.00
30%-3 h	84.72 ± 2.95	62.73 ± 0.07	59.16 ± 4.20	38.11 (5 min) 28.81 (10 min)	27.74 ± 8.28	28.46 ± 9.22	56.20 ± 3.80
30%-3 h-DMSO	N.A	N.A	101.67 ± 7.08	92.06 (5 min) 82.95 (10 min) 71.68 (30 min)	–	–	–

Data are expressed as an average ± standard deviation, N = 9.

### Wettability

The contact angle measurement was performed to evaluate the wettability of the developed biopolymer, and the results showed that all samples had similar contact angles ranging from 54° to 59°, regardless of the zein content (as shown in Table 1). The contact angle of the developed conjugate was comparable to pure zein, which had a measured value of 58.97° ± 10.84, consistent with literature reports (60° water contact angle (θH_2_O^42^)). Flax mucilage had a contact angle of 72.22° ± 3.21, indicating less wetting ability than zein and its conjugates. However, flax mucilage exhibited an increase in wetting behavior over time, with the contact angle dropping rapidly from 72.22° ± 3.21 and the sample dissolving in 10 min, confirming its high water solubility. Sample 30%-3 h had a contact angle of 59.16° ± 4.20, which dropped to 38.11° in 5 min and then reached a plateau at 28.81° in 10 min. Treatment of sample 30%-3 h with DMSO increased the contact angle to 101.67° ± 7.08 and showed more stability over time, with a contact angle of 71.68° measured at t = 30 min (where a plateau is reached).

To determine the free surface energy of the developed conjugates, contact angle measurements were carried out using both water and 3,3′-diiodomethane (DIM). Based on the contact angles observed, the surface energy was calculated. Interestingly, the flax mucilage displayed the highest surface energy (95.3 ± 1.8^43^), which decreased to 48.70 ± 6.02 upon reaction with 5% zein. Surface energy is directly associated with the strength of bulk interactions and the level of surface moieties exposure. Hence, a higher surface energy value indicates greater exposure of functional moieties on the surface. The strong molecular attraction observed for the flax mucilage makes it easier to bond with the available –NH moieties of the zein protein. Moreover, the results indicate an increasing trend in surface energy with the increase in zein content, which could be an indication of excess unreacted –NH moieties being available.

### Morphological analysis

**Figure 2:**
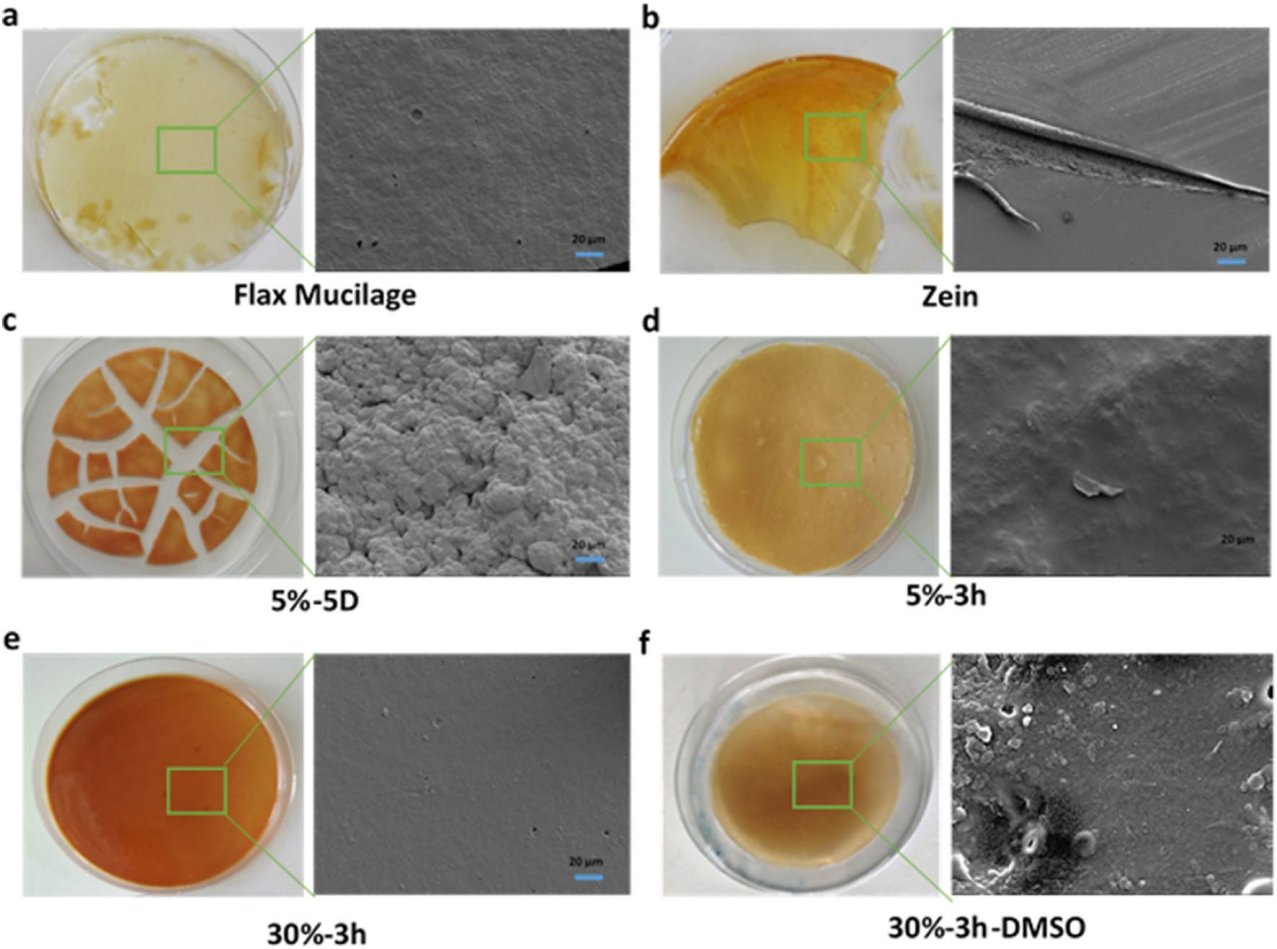
Photographs and SEM micrographs (×500) of the solvent cast (**a**) neat flax mucilage, (**b**) zein, and the different flax mucilage/zein conjugates (**c**) 5%-5D, (**d**) 5%-3 h, (**e**) 30%-3 h, and (**f**) 30%-3 h-DMSO respectively.

Morphological analysis was conducted using direct physical observation and SEM. The dry extracted flax mucilage formed a light brown brittle flaky film, as shown in Fig. 2a. The zein, on the other hand, formed a stiff film with a smooth homogeneous surface, as depicted in Fig. 2b. To investigate the effect of the different modification conditions on the surface morphology, the morphological analysis of 5%-5D, 5%-3 h, 30%-3 h, and 30%-3 h-DMSO was carried out. The SEM micrograph in Fig. 2a shows that the dried flaky flax mucilage film had a very smooth surface. In contrast, the neat zein film exhibited excellent film-forming properties as compared to the flax mucilage and had a smooth homogenous surface with fissures that tend to furl upon closer examination (Fig. 2b).

Significant differences in the surface morphology were observed in all flax mucilage/zein conjugates compared to the neat zein and flax mucilage. The SEM micrographs of 5%-5D revealed an extremely rough, foamy, and textured surface, with predominant cracks on the surface, indicating its brittleness (Fig. 2c). In contrast, the 5%-3 h and 30%-3 h samples displayed extremely smooth and crack-free surfaces, as shown in Fig. 2d,e. This clearly illustrated that the longer the reaction time, the rougher and more textured the samples became, likely due to the high-water holding capacity of the long reaction time samples, which could cause a foaming effect, leading to texture and roughness of the sample and causing cracking upon drying. Furthermore, in the case of the DMSO post-treated samples, the morphology tended to be rougher compared to the untreated (Fig. 2f), likely due to the DMSO evaporation during drying.

### The molecular weight

The molecular weight averages of both 30%-3 h and 30%-3 h-DMSO were determined by GPC and are summarized in Table 2. The GPC curves, which can be found in the supplementary data S1, displayed unimodal symmetric distributions, suggesting that the interaction between the polysaccharide and protein was well controlled. The M_w_ of the biopolymer was found to be lower than the M_w_ of the flax mucilage (1.3 × 10^6^ g/mol) and higher than that of zein (20 KDa), indicating the bonding of flax mucilage and zein protein, resulting in M_w_ of 3.7 × 10^4^ g/mol and 2.9 × 10^4^ g/mol for 30%-3 h and 30%-3 h-DMSO, respectively.

**Table 2:** Molecular weight averages and thermal properties of 30%-3 h, and 30%-3 h-DMSO.

Sample	Molecular weight averages(g/mol)						Thermal properties			
	M_p_	M_n_	M_w_	M_z_	M_z+1_	M_v_	PD	T_g1_	T_g2_	T_c_
Zein	–	–	–	–	–	–	–	94.0 ± 1.9	155.9 ± 0.4	–
Flax mucilage	–	–	–	–	–	–	–	67.8^43^	–	208.31^43^
5%-5D	–	–	–	–	–	–	–	60 ± 2.0	–	172 ± 2.3
30%-3 h	33,754	19,099	37,515	63,709	95,206	59,481	1.96	62.3 ± 2.1	103.1 ± 9.8	180.0 ± 2.0
30%-3 h-DMSO	31,694	19,601	29,998	42,671	56,144	40,752	1.53	49.0 ± 1.8	93.9 ± 11	174.4 ± 9.9

Data are expressed as an average ± standard deviation, N = 3.

The polydispersity of 30%-3 h and 30%-3 h-DMSO were determined to be 1.96 and 1.53, respectively. The results indicate that the mass average molecular weight (M_w_) and polydispersity index (PDI) of the 30%-3 h-DMSO were smaller than those of 30%-3 h. This suggests that the molecular chain length of 30%-3 h-DMSO was shorter and the molecular weight distribution was narrower compared to 30%-3 h. The treated DMSO samples hence resulted in a less heterogeneous polymer with homogenous properties.

### Thermal properties

To assess the thermal properties of the developed conjugates, differential scanning calorimetry (DSC) was carried out. The results of the DSC analysis for the 30%-3 h and 30%-3 h-DMSO samples (see S2) are summarized in Table 2, which also highlights the effect of DMSO treatment on the thermal properties of the conjugate. Three characteristic peaks, typical of polysaccharide DSC plots, were observed for all films. During the first heating cycle, an endothermic peak was noted at around 100 °C, which can be attributed to the loss of trapped moisture within the sample^44,45^. To confirm that the observed endothermic peak was due to water vaporization, the samples were cooled down to − 80 °C and then heated again. In the second heating cycle, the absence of an exothermic peak confirmed that the endothermic peak at 100 °C was related to the evaporation of water trapped in the sample. The films exhibited two glass transition temperatures (T_g1_ and T_g2_) at 62.3 ± 2.1 °C and 103.1 ± 9.8 °C, and 49.0 ± 1.8 °C and 93.9 ± 11 °C for the 30%-3 h and 30%-3 h-DMSO samples, respectively. The samples demonstrate a decrease in T_g_ as the reaction time increases and in the case of DMSO-treated samples. This decrease in T_g_ suggests the restrained retrogradation due to the stronger hydrogen bonds, which leads to the formation of more amorphous structures in these conjugates.

### Physicochemical analysis

**Figure 3:**
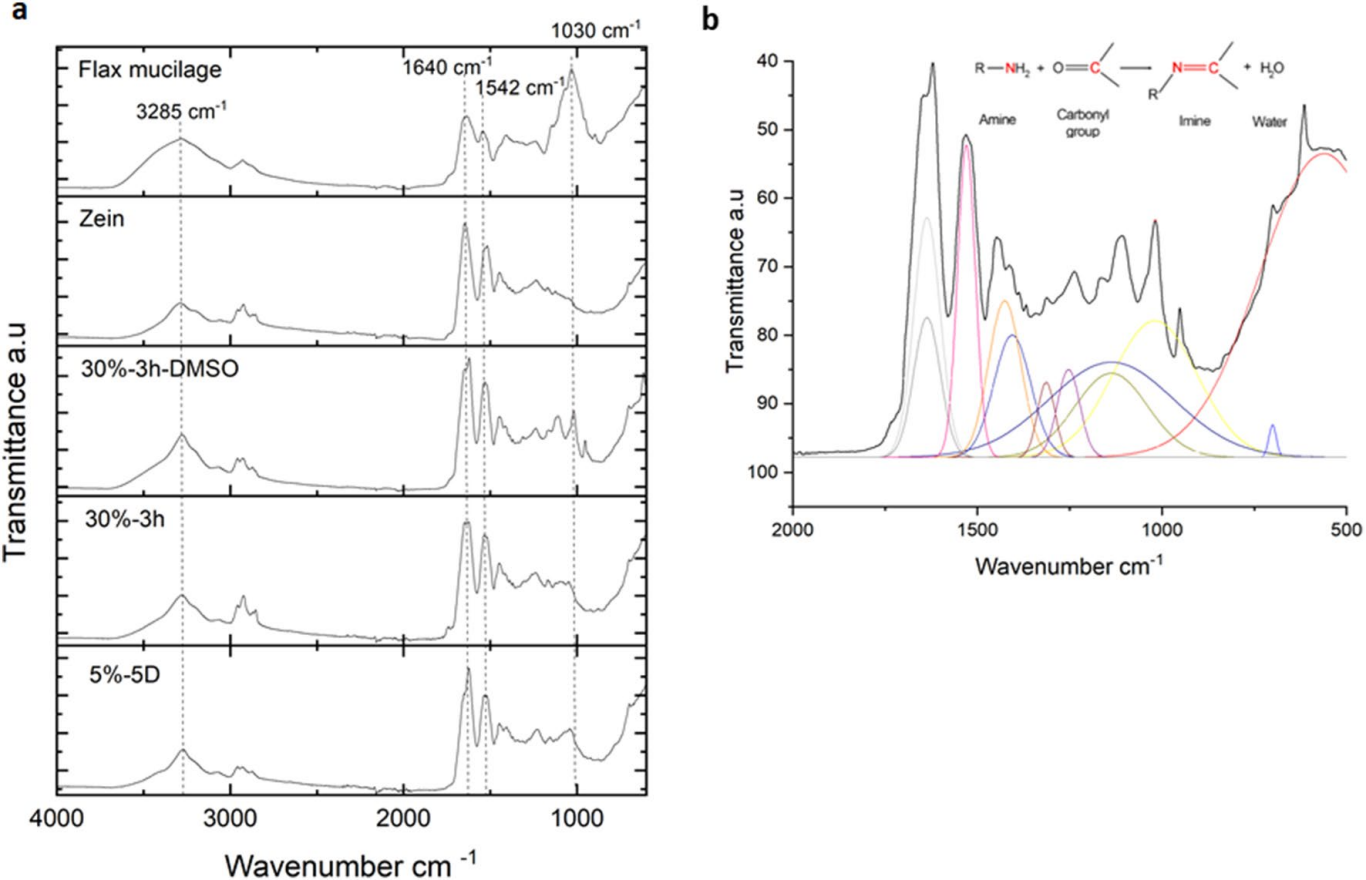
FTIR spectra. (**a**) In the range 4000–500 cm^−1^ of the flax mucilage, zein, 30%-3 h-DMSO, 30%-3 h and 5%-5D indicating the amide I absorptions and the absence of the pyranose at 1047 cm^−1^, (**b**) Deconvoluted region (2000–500 cm^−1^) of 30%-3 h-DMSO spectrum, showing the formation of imine from an amine and a carbonyl-containing structure.

The physicochemical analysis of the flax mucilage/zein conjugate was conducted via FTIR, and the spectra of the neat flax mucilage, zein, and the modified samples are presented in Fig. 3a The broadband at 3285 cm^−1^ in the spectrum of flax mucilage is related to the OH stretching vibrations of hydroxyl groups present in the carboxylic acids, and hydroxyl groups of the carbohydrate structure of flax mucilage^46^. The peak at 2930 cm^−1^ (stretching and bending vibration of CH_2_) and especially at 1030 cm^−1^ (C–O–C) are characteristic of the polysaccharide backbone of pyranoses^47^. The peak at 1408 cm^−1^ is associated with galacturonic acid, and the peaks at 1640 cm^−1^ (C=O stretch) represent the stretching vibration of amide I. Amide II (1542 cm^−1^) and amide III (1240 cm^−1^) were also present, confirming that flax mucilage possessed some proteins as well^48,49^.

In the spectrum of zein, the stretching of the O–H and N–H bonds of the amino acids is visible at 3289 cm^−1^, and the C–H groups are demonstrated by the peaks between 2995 and 2835 cm^−1^^50^. The stretching of the C=O of carbonyl groups of amide I (1645 cm^−1^) and the N–H bending and C–N stretching of amide II (1537 cm^−1^), as well as the axial deformation vibrations of C–N bonds and C=O bending vibrations of amide III (1240 cm^−1^), represent the amino acid structure^51^.

The spectra of both samples 5%-5D and 30%-3 h, showed all the characteristic peaks of the flax mucilage and the zein protein. The distinctive C–H groups between 2995 cm^−1^ and 2835 cm^−1^ occurred only in the spectrum of zein and were present in the modified samples as well. The O–H stretching had slightly weakened compared to the flax mucilage (3285 cm^−1^) and shifted to 3289 cm^−1^ for the 5%-5D and 30%-3 h. This could be due to the interaction and formation of new hydrogen bonds between the flax mucilage and zein. The amide bonds of the conjugate had also shifted compared to both flax mucilage and zein: Amide I moved from 1640 cm^−1^ (flax mucilage) and 1545 cm^−1^ (zein) to 1622 cm^−1^ and amide II shifted from 1542 cm^−1^ (flax mucilage) and 1537 cm^−1^ (zein) to 1530 cm^−1^. Amide III shifted from 1240 cm^−1^ (flax mucilage and zein) to 1230 cm^−1^, those shifts further implied the chemical interaction between the flax mucilage and zein in the modified sample. The very strong peak at 1047 cm^−1^ related to the backbone of pyranoses in the flax mucilage, had disappeared in the modified samples. This demonstrated a transition from the cyclic pyranose conformation into a linear chain. Moreover, the presence of zein added to the speculation that a nucleophilic reaction of the carbonyl groups (C–O) of the flax mucilage occurred and thus, a covalent bond (Schiff base bond) between the flax mucilage and the zein was formed. This nucleophilic reaction was likely to be an imine (C=N), which was indicated in the FTIR spectra by the presence of the peaks in the range of 1640–1690 cm^−1^. The intensity of the C=O characteristic absorption band of the flax mucilage was weakened in 5%-5D and 30%-3 h spectra which indicates the formation of imine bonds due to the partial consumption of aldehyde groups of the flax mucilage via the reaction with amino groups of the zein^52–54^. The results of FTIR analysis confirmed that Schiff bases (C=N double bonds) were formed between the aldehyde groups of flax mucilage and the free amino groups of zein forming a polysaccharide/protein conjugate^52^. Such a covalent bond was enhanced in the samples which were dissolved in DMSO, indicating an increased rate of the nucleophilic reaction as previously demonstrated^53,55^. The deconvoluted region (2000–500 cm^−1^) of 30%-3 h-DMSO FTIR spectrum, shows the formation of imine from an amine and a carbonyl-containing structure (Fig. 3b). The dissolution of anionic reactants in a dipolar aprotic solvent such as DMSO accelerates the rate of amide hydrolysis, as solvation increases the activity of the anionic reactants^53,55^.

### NMR analysis

**Figure 4:**
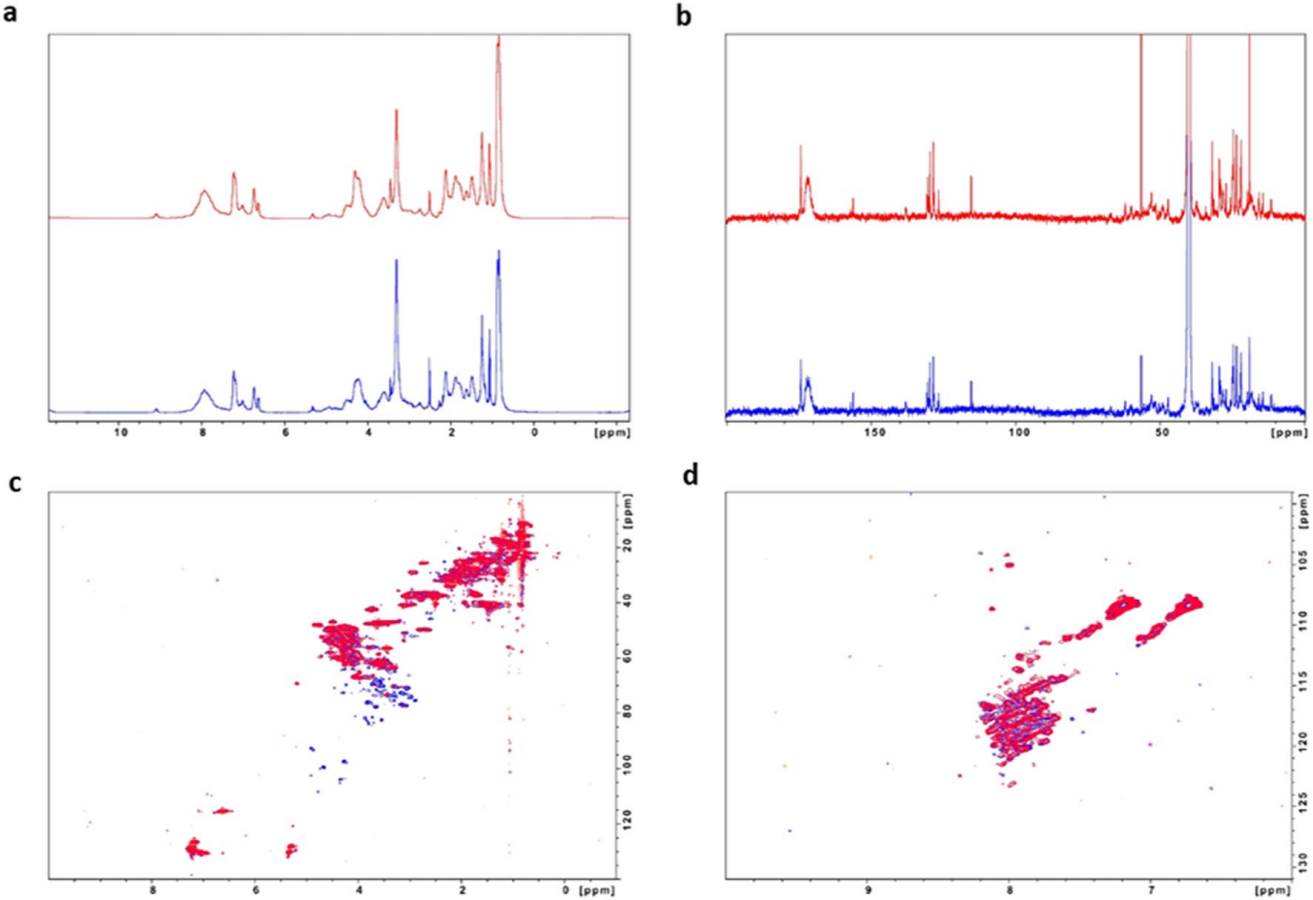
NMR spectra (**a**) 1D ^1^H NMR, (**b**) 1D ^13^C NMR, (**c**) 2D ^1^H, ^13^C-HSQC and (**d**) 2D ^1^H, ^15^N-HSQC spectra (red: 30%-3 h and Blue: 5%-5D).

In this study, it is suggested that a glycation process occurred between the flax mucilage and zein, resulting in the formation of an advanced glycation end product (AGE). To confirm this, NMR spectroscopy was carried out on the 5%-5D and 30%-3 h samples. The 1D ^1^H NMR spectra of both samples were similar and indicative of a large protein with poorly resolved and broad signals, which can be attributed to a large molecular size or sample aggregation (Fig. 4a). The 1D ^13^C NMR spectra of the 5%-5D and 30%-3 h samples both showed signals at 67.9 ppm, 72.2 ppm, and 72.3 ppm, which were attributed to the C–H group of rhamnose and mannose (Fig. 4b). The signals at 130 ppm were attributed to aromatic carbons, while the overlapping peaks around 68–78 ppm were attributed to aliphatic carbons. The signal at 170 ppm was related to the carbonyl group (C=O) of the zein.

The 2D ^1^H,^13^C-HSQC NMR spectra of both samples revealed the presence of a sugar composition. The signals between δ(^1^H) = 3.58–3.40 ppm could be attributed to the C–H and O–H group of rhamnose, mannose, and arabinose, while the signal at 1.25 ppm was assignable to the methyl group (CH_3_) of rhamnose residues (Fig. 4c). The chemical structure of flax mucilage polymer after modification with zein showed obvious changes as illustrated by 2D ^1^H,^13^C-HSQC NMR spectroscopy (Fig. 4c). The signals at 20 and 50 ppm were related to the hydrocarbons present in the flax mucilage, such as the CH_3_ peaks at δ = 23.5 and 27.8 ppm, the CH_2_ peak in the aliphatic part at δ = 45.2 ppm, and the C–H signals at δ = 26.9 and 32.3 ppm. The appearance of new peaks at δ = 155–157 ppm was attributed to imine (C=N) linkages. The reaction between the –NH group from zein and the –OH groups from flax mucilage polymer was evidenced by the appearance of a new peak corresponding to imine (C=N) linkages at 155.87 in the flax mucilage/zein conjugate complex.

Furthermore, the 2D ^1^H,^13^C-HSQC spectra showed significant differences in the regions 3–4 ppm/70–80 ppm (sugar region) and 4–5 ppm/97.5–103.3 ppm correlating with the anomeric carbons of the flax mucilage. A signal at 5.25 ppm, whose carbon atom resonates at 100.2 ppm, was assigned to the rhamnose residues of a rhamnogalacturonan backbone (see Fig. 1a). Another common signal observed in NMR spectra for different mucilages is a proton, in that case, we see a signal at 4.94 ppm correlating with a carbon atom at 100.4 ppm, presumably due to the galacturonic acid residues of the polysaccharide. In terms of signal intensity, the 5%-5D (blue spectra) showed more diverse signals in some regions, while in other regions, the 30%-3 h signals (red signals) were ca. 20% stronger. The glycosylation state of the proteins appeared to be different, as indicated in Fig. 4c, where the content of zein and the reaction time altered the glycosylation state.

The 2D ^1^H,^15^N-HSQCs spectra (Fig. 4d) showed characteristic protein signals, with the Asn and Gln side chains appearing as two distinct "ridges" between 6.5 and 7.5 ppm/110 and 112 ppm, respectively. The H–N groups were represented by a broad "blob" of overlapping signals centered around 8 ppm/117 ppm. However, the signals were weak and poorly dispersed, suggesting that the zein protein had been denatured or unfolded. The NMR findings were consistent with the FTIR results, providing further evidence that the flax mucilage and zein had undergone a Maillard reaction to form a polysaccharide–protein conjugate.

### Solubility study

**Figure 5:**
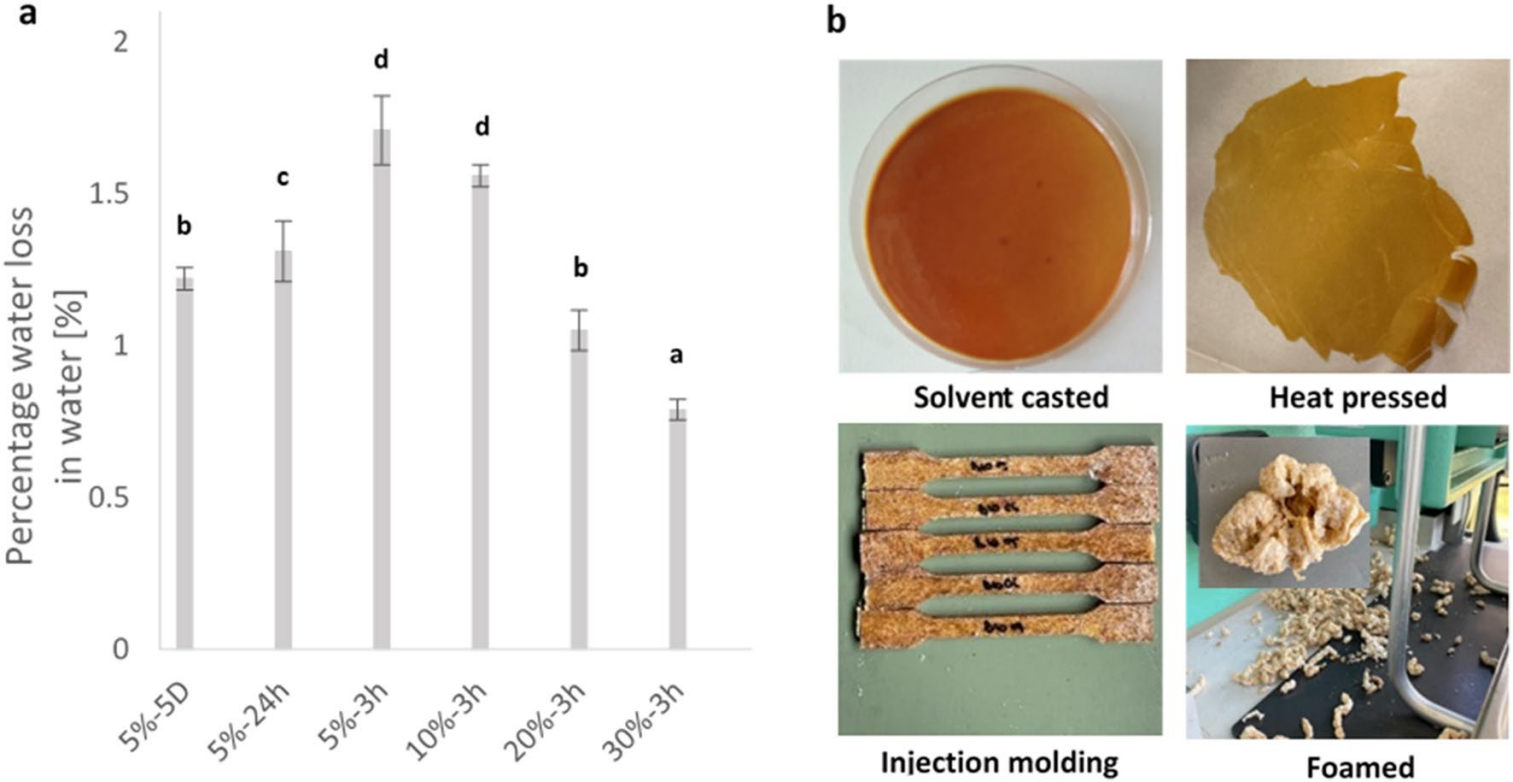
(**a**) A qualitative solubility study showing the percentage weight loss of the different samples, bars with different letters indicate significant difference in percentage water loss (P < 0.05). (**b**) 30%-3 h sample through different processing techniques. The solubility study were conducted in triplicate, with each set of measurements performed three times (data are expressed as an average ± standard error, N = 9).

Despite the high affinity of the samples for water, they exhibited low solubility and absorption of water after drying. To evaluate their solubility, a qualitative solubility study was conducted on the samples. The results indicated the highest weight loss for 5%-3 h with 1.71 ± 0.34% and the lowest for the 30%-3 h with 0.79 ± 0.1% in 24 h (Fig. 5a). Moreover, the stability of the 30%-3 h sample in various solvents was tested quantitatively. The results revealed that the 30%-3 h sample was soluble in DMSO but resistant to ethanol, methanol, acetone, and toluene (refer to supplementary data S3 for more information).

### Processability

Figure 5b illustrates how the flax mucilage/zein conjugate can be used in various processing techniques. The solvent casting technique was used to prepare thin films. The heat press method was employed to produce thicker samples with controlled dimensions. Injection molding was utilized to produce samples with specific shapes, while the foam technique was employed to produce a lightweight structure. These results highlight the potential of the flax mucilage/zein conjugate as a renewable and biodegradable material with broad applicability in various conventional processing techniques.

### Mechanical properties

**Figure 6:**
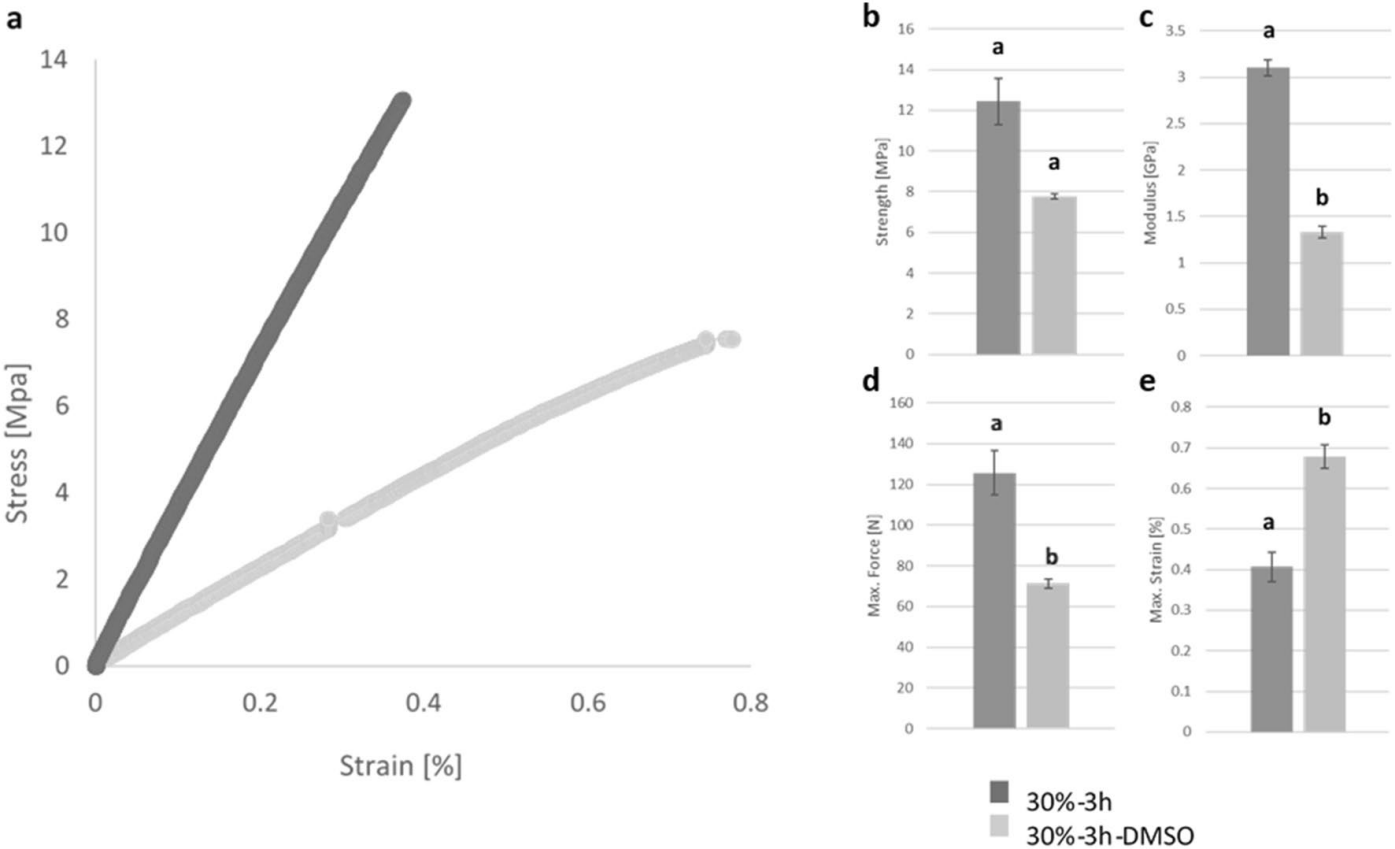
(**a**) Stress–strain cure for 30%-3 h and 30%-3 h-DMSO. (**b**) Strength, (**c**) E-modulus, (**d**) Max. Force (**e**) Max. Strain, bars with different letters are significantly different (P < 0.05). The the mechanical tests were conducted in triplicate, with each set of measurements performed three times (data are expressed as an average ± standard error, N = 9).

The mechanical properties of the flax mucilage/zein compared to neat flax mucilage and zein, showed an increase in Young’s modulus from 0.31 ± 0.02 GPa^43^ and 0.409 ± 7.62 GPa^56^ respectively to 3.10 ± 0.26 GPa confirming the successful conjugation process. The tensile testing results in Fig. 6a and Table 3 confirmed that the M_w_ played a core role in the mechanical properties of the flax mucilage/zein conjugate. The 30%-3 h showed higher Young’s modulus, tensile strength and max force compared to the 30%-3 h-DMSO (Fig. 6b–d) which might be explained by the higher M_w_ of the 30%-3 h sample. However, the elongation at break slightly increased from 0.41 ± 0.11% to 0.68 ± 0.09% corresponding to 30%-3 h and 30%-3 h-DMSO samples, respectively (Fig. 6e), demonstrating the plasticizing effect of the DMSO post-treatment.

**Table 3:** Summaries the mechanical of the different 30%-3 h and 30%-3 h-DMSO samples.

Name	Max. force (N)	Strength (MPa)	E-modulus (GPa)	ε_max_ (%)
Zein	**–**	6.70 ± 0.37^56^	0.409 ± 7.62^56^	1.96 ± 0.18^56^
Flax mucilage	**–**	16.46 ± 1.08^43^	0.31 ± 0.02^43^	55.68 ± 2.11^43^
30%-3 h	125.72 ± 32.61^a^	12.43 ± 3.42	3.10 ± 0.26^a^	0.41 ± 0.11^a^
30%-3 h-DMSO	71.0 ± 6.64^b^	7.77 ± 0.36	1.33 ± 0.20^b^	0.68 ± 0.09^b^

Data are expressed as an average ± standard error, N = 9. Different letters in a column indicate significant differences in the percentage of Max. Force, strength, E-modulus, and Max. Strain among the different samples (p < 0.05) (data analyzed with one-way ANOVA and Tukey’s post hoc test).

## Discussion

Cultivating flax solely for flax mucilage extraction to produce the flax mucilage/zein conjugate bioplastic would not be environmentally sustainable. However, flax is already cultivated for its seed oil and linen, and flax mucilage is a by-product that can be extracted from the waste flax seed cake generated by the flax oil extraction industry, thereby adding value to an otherwise wasted material. When performing a life cycle analysis (LCA), this should be taken into consideration for better waste management and the replacement of oil-based plastic packaging. Additionally, zein is a byproduct of starch or oil extraction from corn meal. LCA of other biodegradable plastics based on proteins has shown that they have a positive impact at the end of their life cycle in a composting scenario. Such materials integrate quickly into the soil through microorganisms, reducing the amount of waste sent for incineration and thereby minimizing the associated emissions^57^.

In this study flax mucilage and zein underwent a glycation process, resulting in the formation of an AGE confirmed by NMR analysis. The glycation process for any polysaccharide/protein conjugate begins with the formation of a Schiff base, leading to the production of an intermediate Amadori product and other intermediate compounds. When the Amadori adduct undergoes oxidation, it forms an advanced glycation end product. This glycation process is also known as non-enzymatic glycosylation or Maillard reaction^58^. All reducing sugars can undergo a glycation reaction with various amino acids^58,59^. One of the main factors that affect the rate of the Maillard reaction is the type of sugar used. For instance, pentoses tend to be more reactive than hexoses in general. According to previous studies, the glycation ability of pentoses was observed to increase in the following order: d-glucose <  d-mannose <  d-galactose <  d-xylose <  d-fructose <  d-arabinose <  d-ribose^59,60^. d-Ribose is the most reactive in the glycation of proteins and results in more rapid production of AGEs than other reducing sugars in-vitro and in-vivo^59^.

A glycation end product via Maillard conjugates in general can be prepared from conventional or novel methods, each has its advantages and limitations. Here we represented a simple and non-toxic method for the development of flax mucilage/zein conjugate^61,62^. The developed conjugate after appropriate glycation showed enhanced functional properties, less solubility, thermal stability, foaming capacity and higher mechanical performance. However, research on the structure–function relationship for both proteins and polysaccharides is limited thus, it is necessary to understand and characterize the developed conjugates and select appropriate conditions to control and optimize the process of the Maillard reaction. In other studies, Maillard conjugates showed great potential as emulsifiers and stabilizers in emulsion systems^63^, while the present study shows its potential to form functional conjugates as a biogenic thermoplastic alternative.

The DMSO treatment affected the physicochemical, thermal, and mechanical properties of the flax mucilage/zein conjugate. With optical observation, the 30%-3 h-DMSO samples appeared less opaque and had slight transparency compared to the 30%-3 h samples which might be explained by the polymer crystallinity. The contact angle changes were a function of polymer crystallinity, surface energy, functional groups present on the surface, surface heterogeneity, and roughness^64,65^. Upon the treatment of the 30%-3 h sample with DMSO the contact angle increased which could be due to the increase in roughness of the surface as confirmed by the SEM micrographs in Fig. 2f. The DMSO treatment affected the thermal properties of the developed conjugates, lowering both their T_g1_ and T_g2_. This is explained by the lower M_w_ of the 30%-3 h-DMSO sample, as it is known that the T_g_ increases with an increase in the M_w_ and asymptotically approaches a maximum value^66^. In addition, a higher Young’s modulus was attributed to the increase in the chain length and higher M_w_. Thus one can modify the surface structure, crystallinity, thermal properties, and mechanical properties of the developed flax mucilage/zein conjugate by altering its molecular weight or destruct the crystalline phase and transferring the conjugate into a continuous amorphous structure using a plasticizer such as DMSO.

The developed flax mucilage/zein conjugate exhibits significant brittleness, with a strain value of less than 1%. This level of brittleness renders it unsuitable for direct use in packaging applications. However, it is worth noting that the addition of additives and plasticizers has the potential to enhance its mechanical properties and mitigate this brittleness issue. In future research, a thorough investigation and optimization process will be carried out to identify and optimize the ideal additives and plasticizers that can effectively mitigate the brittleness issue of the developed flax mucilage/zein conjugate.

## Conclusion

Flax mucilage was successfully extracted from flaxseed cake residue using a simple and non-toxic method, resulting in a satisfactory yield ranging 75–84%. Subsequent glycation with zein yielded a functional conjugate functional polymer with promising thermoplastic properties, exhibiting Young's modulus of 1–3 GPa. The developed conjugate showed improved functional characteristics, including enhanced thermal stability, reduced solubility of less than 1%, increased foaming capacity, and superior mechanical performance. However, there is still limited research on the structure–function relationship of both proteins and polysaccharides, emphasizing the importance of further characterization of the developed conjugates and optimization of the Maillard reaction process conditions. The developed polymer showed its potential to be processed via various methods such as solvent casting, injection molding, or puffed into foams. Although further studies are needed to explore the use of plasticizers to enhance the brittleness of the produced films, the fabricated flax mucilage exhibits promising potential as a candidate for various industrial applications. Furthermore, future investigations will focus on the determination of the barrier properties, as well as the degradation kinetics of this novel conjugate material.

## Materials and methods

Flaxseed cake from residues was kindly provided by Lausitzer Ölmühle (Hoyerswerda, Germany). Ethanol (≥ 99.8%, denatured) was bought from Carl Roth (Germany). All other chemicals were bought from Sigma Aldrich (Germany); potassium persulfate (ACS reagent, ≥ 99.0%), DMSO (anhydrous, ≥ 99.9%), DIM (analytical standard), *N*,*N*-dimethylformamide (DMF) (HPLC grade), lithium bromide (*ReagentPlus*^®^, ≥ 99%), DMSO-d6 (deuteration degree min. 99.95% for NMR spectroscopy MagniSolv™), acetone (ACS reagent, ≥ 99.5%), toluene (ACS reagent, ≥ 99.5%).

### Flax mucilage extraction

The flax mucilage extraction was carried out using the hot water method with slight modification as described in^43^. In summary, the flaxseed cake was soaked in water (with a seed-to-water ratio of 1:30) at 60–90 °C for different durations (1–6 h) under constant stirring. Subsequently, the suspension was centrifuged at 3000 rpm for 20 min to remove the seeds from the extracted mucilage. The extracted mucilage was precipitated using two volumes of 98% ethanol. After a period of 1 h at 25 °C, the precipitate was collected by centrifugation at 3000 rpm for 20 min. The precipitated solid was dried in a hot air oven at 45 °C for 24 h and ground to a fine powder, the obtained yield was calculated in relation to the initial weight of the flax cake used.

1 $$\frac{\mathrm{Extracted \, mucilage \, weight}}{\mathrm{Flax \, seed \, weight}}\times100=\% Yield$$

### Development of flax mucilage/zein bioplastic

The extracted flax mucilage was added to a mixture of ethanol/water (50:50) to a concentration of 1.65% w/v% (16.5 g/L). Different flax mucilage solutions were prepared with different zein content concentrations varying between 5 and 30% w/v%. In addition, 0.8% w/v% potassium persulfate (K_2_S_2_O_8_) was added as a prominent oxidizing agent to activate functional groups and initiate grafting polymerization of zein onto the flax mucilage polysaccharide backbone as seen in Table 4. Moreover, sample 30%-3 h was treated with DMSO to study the effect of a dipolar aprotic solvent on the morphological, thermal and physicochemical properties of the conjugate.

**Table 4:** Different flax mucilage/zein biopolymers of different zein content, oxidizing agent, and reaction time.

Samples	Flax mucilage solution (16.5 g/L) (mL)	Zein (g)	Zein (w/v%)	K_2_S_2_ O_8_ (g)	Time (h)
5%-5D	1000	50	(5%)	8	120
5%-24 h	1000	50	(5%)	8	24
5%-3 h	1000	50	(5%)	8	3
10%-3 h	1000	100	(10%)	8	3
20%-3 h	1000	200	(20%)	8	3
30%-3 h	1000	300	(30%)	8	3
30%-3 h-DMSO	Sample 30%-3 h is treated with DMSO (1:10) for 120 days, then dried and degassed				

For all the upcoming analytical analyses, solvent casted samples were prepared using either water or dimethyl sulfoxide (DMSO) as the solvent. The preparation process involved dissolving the materials in 10 mL of the respective solvent and subsequently casting them into an aluminum petri dish. The samples were then dried in a hot air oven set at 50 °C until complete evaporation of the solvent occurred.

### Contact angle measurement

Contact angle measurements were carried out to determine the wettability and the free surface energy of the developed flax mucilage/zein conjugates. The wettability was evaluated using a static contact angle measurement device, Drop Shape Analyzer DSA25 (Krüss GmbH, Germany). Deionized water (3 μL) and DIM were used as the reference liquids using a gas-tight micro-syringe. Three repetitions of each reference liquid for each sample were carried out for three sets of samples, N=9. The θH_2_O were measured by analyzing the recorded drop images using the Drop Shape Analysis 1.0 software after 5 s.

### Scanning electron microscopy

The surface morphology of the developed flax mucilage/zein conjugates was conducted using conventional SEM**,** JSM-7200F (Jeol Ltd., Tokyo, Japan) at 1 kV, under high vacuum. For the sample preparation, the samples were dried additionally for 24 h in a hot air oven at 50 °C to decrease the moisture content. Then the samples were fastened on a double-sided adhesive carbon tape and sputtered with gold.

### Gel permeation chromatography

4 mg of each sample were dissolved in DMF containing 25 mmol/L of lithium bromide (LiBr), to a concentration of 2 mg/mL. Subsequently, the samples were subjected to an ultrasonic treatment bath for approximately 4 h. The solutions were then filtered through 0.45 µm PTFE filters and subsequently analyzed using the 1260 Infinity II GPC/SEC System (Agilent Technologies, Germany). The measurements were conducted at a temperature of 40 °C, employing refractive index detection with respect to PolarGel-M columns and the light scattering measurements were performed at 620 nm and angles of 15° and 90°.

### Differential scanning calorimetry

The glass transition temperature (T_g_) of the developed flax mucilage/zein conjugates was recorded with a TA Instrument, Q200 DSC. For each sample, approximately 5 mg were subjected to two heating runs from − 80 to 120 then cooled down to − 80 followed by a second heating run from − 80 to 210 °C at a heating rate of 20 °C min^−1^.

### Fourier-transformed infrared spectroscopy

The developed flax mucilage/zein conjugate was analyzed by ATR-FTIR spectroscopy with SpectrumTwo™ (PerkinElmer, Inc, Waltham, USA) recording 25 scans with 4 cm^−1^ resolution in the range of 4000–500 cm^−1^. Both neat extracted flax mucilage and zein were examined for comparison.

### Nuclear magnetic resonance spectroscopy

The flax mucilage/zein was analyzed by solution-state NMR spectroscopy on a 500 MHz Bruker Avance III spectrometer (Bruker BioSpin GmbH, Ettlingen, Germany), equipped with a triple-resonance helium-cooled cryoprobe and running the Bruker Topspin3.5pl7 software package. Samples were prepared by manually crushing ca. 50 mg in a mortar and treating the coarse powder with 0.6 mL of DMSO-d6 at room temperature overnight, then using the clear, slightly yellowish supernatant. ^1^H spectra were run with 128 scans/16,384 real data points, 2D ^1^H, ^13^C-HSQC spectra (Bruker library pulse program “HSQCETGP**”**) with 16 scans and 256 t_1_ increments/2048 data points in t_2_, 2D ^1^H, ^15^N-HSQC spectra (pulse program hsqcetf3gp) with 16 or 32 scans and 256 t_1_ increments/2048 data points in t_2_; 1D ^13^C spectra (pulse program zgig30) were acquired with 4096 scans/16,384 data points. All spectra were run at 308 K sample temperature.

### Solubility study

To conduct the solubility test and monitor weight loss, various flax mucilage/zein conjugate samples were tested in water over a 24-h period (namely: 5%-3 h, 5%-48 h, 5%-5D, 10%-3 h, 20%-3 h, and 30%-3 h). Each sample, weighing 1–2 mg, was initially dried for 24 h at 50 °C and weighed (W_Dry 1_). Subsequently, the samples were immersed in water at room temperature and periodically stirred slowly with a flat shaker for 24 h. Later, the sample was removed and dried for 24 h at 50 °C. The resulting samples were weighed (W_Dry 2_), and the solubility was calculated using the following equation:

2 $$\% \, Solubility=\frac{{W}_{Dry 1}-{W}_{Dry 2}}{{W}_{Dry 1}}\times 100$$

In addition, a qualitative evaluation of the samples was performed by immersing 1–2 mg of the sample in 10 mL in different solvents; water, acetone, toluene, ethanol and DMSO. The solubility of the samples in the various solvents was determined via visual observation over 14 days.

### Processability

To prepare the samples using solvent casting, 10 mL of either water or DMSO was used to dissolve the sample, which was then cast into an aluminum petri dish. The dish was left to dry for 5 days at a temperature of 50 °C. For the heat-pressed samples, a pressure of 5 tons and a temperature of 80 °C were applied to a dry sample using a Biorad Min Lab press. The injection molding process involved injecting the sample at a temperature of 120 °C and a mold temperature of 80 °C. For the foam technique, the sample was extruded at 120 °C.

### Universal tensile testing machine

The tensile testing samples were prepared to dimensions of 30 mm × 6 mm × 1.5 mm and were subjected to 3-day drying in a vacuum oven before testing. Tensile tests were performed following DIN EN ISO 527-4 using a UTM Inspekt 250 machine (Hegewald & Peschke, Germany). The force was measured via a 500 N load cell, and tests were executed at a constant loading rate of 0.5 mm/min with a span of 20 mm. The Youngs modulus was determined from the stress/strain curve between elongations of 0.05% and 0.25%. All measurements were taken under controlled environmental conditions of a temperature of 25.3 ± 0.33 °C and a humidity of 20.9 ± 2.9%. Nine samples were tested for each sample type.

### Statistical analysis

The experimental setup was conducted in triplicate, with each set of measurements performed three times (N = 9) and the results expressed as an average ± standard deviation. Data and Charts were drawn and analyzed using OriginPro, Version Number (Version 2023b) (OriginLab Corporation, Northampton, MA, USA). The results were statistically analyzed using one-way ANOVA with Tukey’s post hoc test, the significant level was set at P < 0.05. All measurements of SEM, DSC and FTIR were conducted in triplicate. After confirming the similarity among the triplicate measurements, one sample from each set was selected for presentation. NMR and GPC were conducted once for each sample tested.

### Supplementary Information

Supplementary Information.

## Data Availability

Correspondence and requests for materials should be addressed to M.T. based on reasonable request.
