# Supplementary material for: A novel polysaccharide/zein conjugate as an alternative green plastic
Source: Sci Rep. 2023 Aug 12;13:13161. doi: 10.1038/s41598-023-40293-4 (PMC10423201; doi:10.1038/s41598-023-40293-4)
Supplement: Supplementary file 1 — Supplementary Information. [file 41598_2023_40293_MOESM1_ESM.docx]

**S1: GPC analysis**

dW/dLogM

| dW/dLogM | Fitted M_w_(g/mol) |
| --- | --- |
| Fitted M_w_(g/mol)  30%-3h | 30%-3h-DMSO |

Fig. S1: Shows the GPC analysis presenting the M_w_ distribution plot for the 30%-3h and 30%-3h-DMSO samples.

**S2: DSC**

| **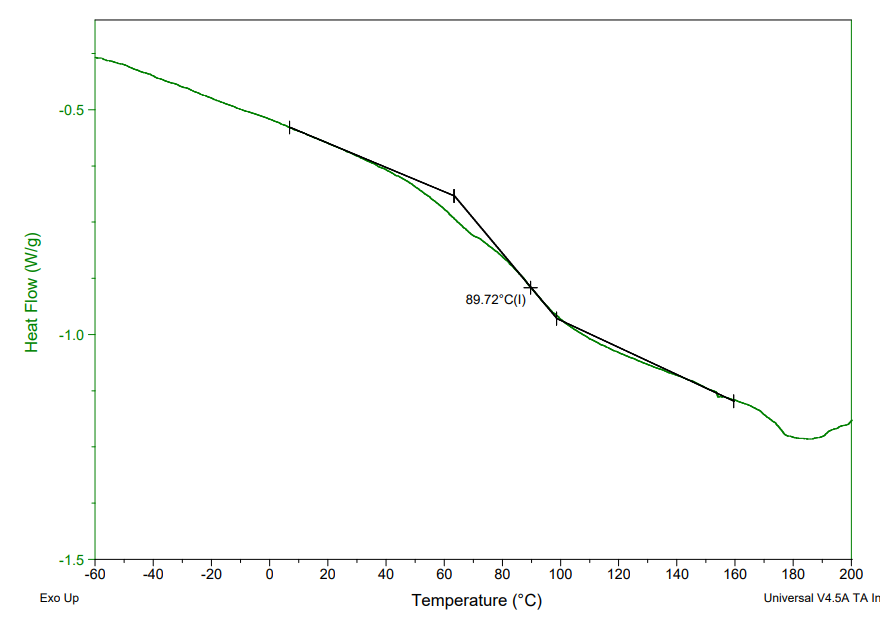** | **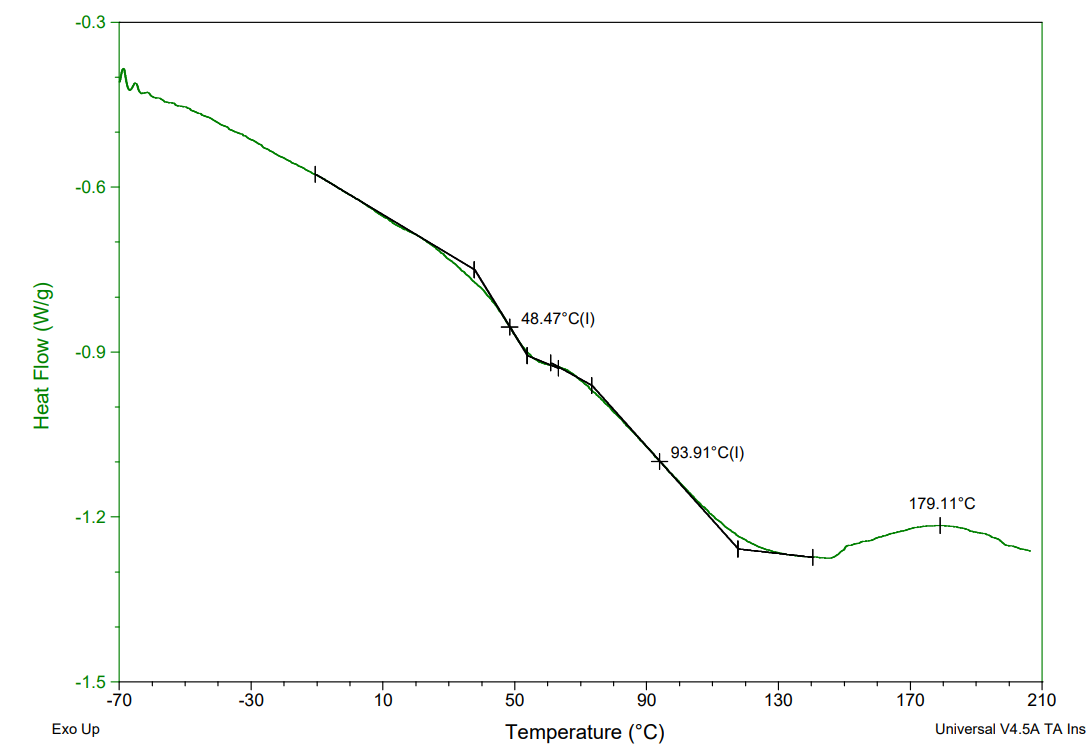** |
| --- | --- |
| 30%-3h | 30%-3h-DMSO |

Fig. S2: Shows the DSC analysis of 30%-3h and 30%-3h-DMSO samples (second heating run).

**S3: Quantitative** **solubility study**

| **Samples** | **Ethanol** | **Methanol** | **Acetone** | **Toluene** |
| --- | --- | --- | --- | --- |
| **30%-3h** | **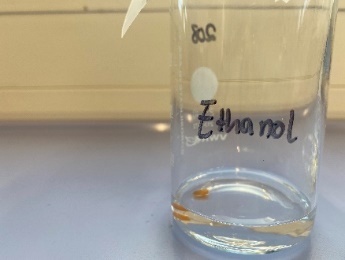** | **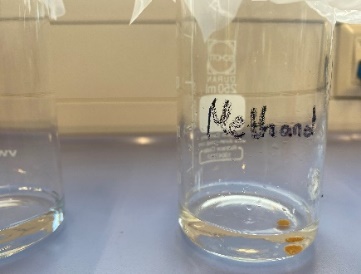** | **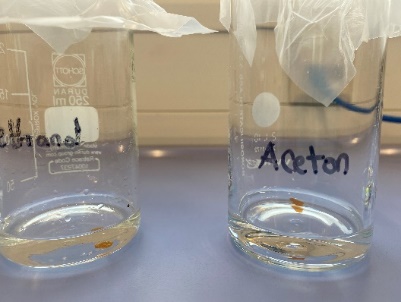** | **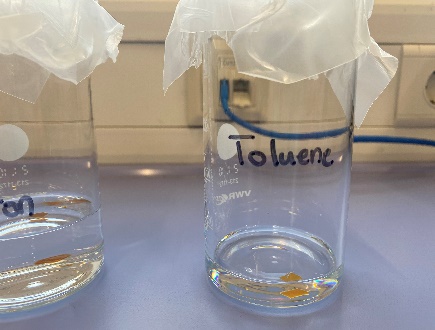** |
| **30%-3h-DMSO** | 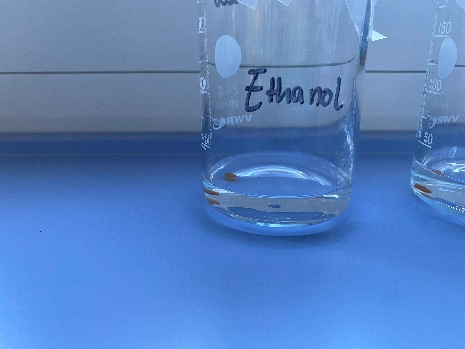 | 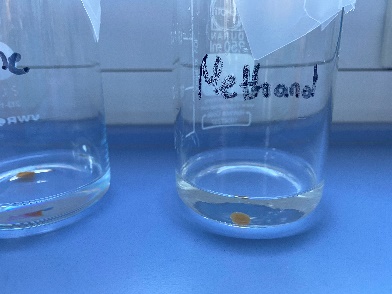 | 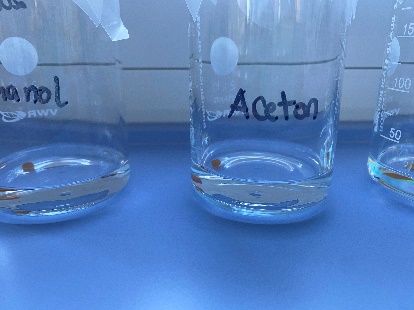 | 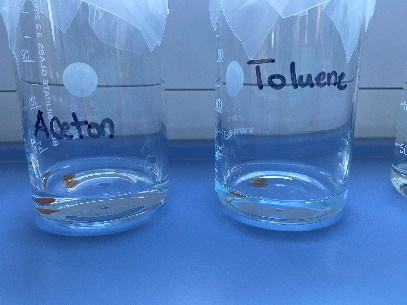 |

Fig. S3: Shows the solubility resistance over 14 days for 30%-3h and 30%-3h-DMSO to different solvents.
